# Extra-cerebral recombination activity of Emx1-Cre and nestin-Cre in the kidney

**DOI:** 10.3389/fcell.2024.1480217

**Published:** 2024-10-16

**Authors:** Min Wang, Xiaojuan Liu, Yin Fang, Qintong Li

**Affiliations:** ^1^ Departments of Laboratory Medicine, Obstetrics & Gynecology and Pediatrics, West China Second University Hospital, Key Laboratory of Birth Defects and Related Diseases of Women and Children, Ministry of Education, Development and Related Diseases of Women and Children Key Laboratory of Sichuan Province, State Key Laboratory of Biotherapy and Collaborative Innovation Center of Biotherapy, Sichuan University, Chengdu, Sichuan, China; ^2^ Department of Laboratory Medicine, West China Second University Hospital, Sichuan University, Chengdu, Sichuan, China

**Keywords:** Emx1-Cre, nestin-Cre, brain, kidney, neurodevelopmental disorders, LARP7, mouse model

## Abstract

Individuals with neurodevelopmental disorders (NDDs) are frequently diagnosed with comorbidities in other organs, indicating that NDD risk genes may have extra-cerebral functions. The engineered mouse models are pivotal in understanding the functions of candidate NDD genes. Here, we report that Emx1-Cre and nestin-Cre mouse strains, the popular tools to study brain development, also exhibit recombination activity in the kidney. We find that both Emx1-Cre and nestin-Cre can drive recombination in epithelial cells lining proximal and distal convoluted tubules of the nephron. Additionally, nestin-Cre drives recombination in the glomerulus of the nephron. Furthermore, we use Emx1-Cre and nestin-Cre to knock out *Larp7*, a gene linked to a human NDD called Alazami syndrome. We find that *Larp7* knockout using nestin-Cre, but not Emx1-Cre, results in elevated blood urea nitrogen. This result suggests a compromised kidney function, reminiscent of recently revealed renal anomalies in Alazami syndrome patients. Many genes have been knocked out using Emx1-Cre and nestin-Cre to study their roles during embryonic neurogenesis. It will be of great interest to reinvestigate whether the renal development and function is affected in these existing mouse models.

## Introduction

Neurodevelopmental disorders (NDDs) are of great societal importance, and remain medically challenging worldwide (Thapar et al., 2017). The fifth edition of the Diagnostic and Statistical Manual of Mental Disorders (DSM-5) categorizes seven disorders under the NDDs umbrella, including intellectual disabilities (ID), autism spectrum disorder (ASD), and attention-deficit/hyperactivity disorder (ADHD). These conditions are characterized by developmental deficits that produce impairments of personal, social, academic, or occupational functioning. Observational epidemiological studies have demonstrated that different NDDs are usually co-occurring with one another (Gidziela et al., 2023). In addition, it is common that individuals with NDDs also have other comorbidities in extra-cerebral organs, such as abnormalities in the urogenital and gastrointestinal systems (Dingemans et al., 2024). Damaging mutations in developmentally important genes makes a major contribution to NDDs (Deciphering Developmental Disorders, 2017). These observations indicate shared etiologies on brain development among NDDs (Parenti et al., 2020), but also suggest that mutations in NDD genes may have pleiotropic effects on extra-cerebral organs (O'Donovan and Owen, 2016). Indeed, human genome sequencing studies have implicated damaging mutations in many candidate genes shared by different NDDs (Gonzalez-Mantilla et al., 2016).

Genetically engineered mouse models generated by the Cre–*loxP* system are instrumental in deciphering the developmental genetics of normal brain development and etiologies underlying NDDs (Kim et al., 2018). Emx1-Cre and nestin-Cre mouse lines are popular tools to knock out gene of interest in the central nervous system (Tronche et al., 1999; Gorski et al., 2002). Emx1-Cre is expressed in neural stem and progenitor cells before embryonic day (E) 10.5, and its activity is primarily localized in the dorsal and medial palliums of the developing neocortex (Gorski et al., 2002). For nestin-Cre, the recombination activity occurs at a later developmental stage than Emx1-Cre. At E12.5, nestin-Cre is highly expressed in differentiating neural cells, but not in neural stem and progenitor cells. The recombination activity gradually peaks in neural stem and progenitor cells between E14.5 and E18.5 (Liang et al., 2012). One advantage of nestin-Cre is its widespread expression in almost all neural cells in the embryonic brain (Tronche et al., 1999). Inducible versions of Emx1-Cre and nestin-Cre are also available, enabling a more precise temporal control of Cre expression (Kessaris et al., 2006; Sun et al., 2014).

In the present study, we show that both Emx1-Cre and nestin-Cre show extra-cerebral recombination activity in epithelial cells in the proximal and distal tubules. Additionally, nestin-Cre drives recombination in the glomerulus of the nephron. Building upon these findings, we use Emx1-Cre and nestin-Cre to knock out *Larp7*, a gene linked to a human NDD called Alazami syndrome (Alazami et al., 2012) (OMIM, # 615071). Interestingly, recent studies have also shown that renal anomalies occur in some patients with Alazami syndrome (Fauntleroy-Love et al., 2023). In this study, we find that *Larp7* knockout by nestin-Cre but not Emx1-Cre may compromise kidney function, suggesting that defects in the glomerulus may underlie the renal anomalies observed in patients.

## Results

### Emx1-Cre mediates recombination in the renal cortex

In our previous studies, we found that knockout of neurodevelopmental genes using Emx1-Cre sometimes exhibited phenotypes that could not be easily explained by compromised brain function. We suspected that Emx1-Cre might mediate recombination in other organs.

**FIGURE 1:**
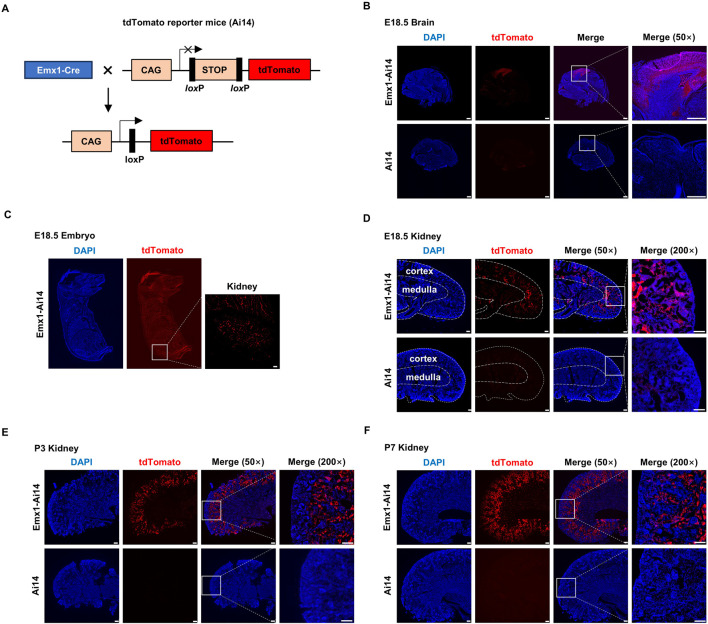
Emx1-Cre mediates recombination in the renal cortex **(A)** The schematic presentation of using tdTomato reporter mice (Ai14) to detect the tissue-specific expression of Emx1-Cre. tdTomato will exhibit red fluorescence in any cells in which Emx1-Cre is expressed. CAG, chicken beta-actin promoter. STOP, STOP cassette containing stop codons in all 3 reading frames and a triple poly(A) signal. Emx1-Cre mice and Ai14 mice were crossed to generate Emx1-Ai14 mice. **(B)** Sagittal view of micro-dissected whole brain from Ai14 and Emx1-Ai14 embryos (n = 3). White boxes were amplified in the right panel. tdTomato was detected in neocortex as expected. Scale bar: 500 μm. **(C)** Sagittal view of E18.5 Emx1-Ai14 embryos (n = 3). White box amplified in the right panel denotes embryonic kidney. Scale bar: 500 μm. **(D–F)** Sagittal view of micro-dissected E18.5 **(D)**, postnatal day (P) 3 **(E)**, and P7 kidneys **(F)** from Ai14 and Emx1-Ai14 mice (n = 9). Areas demarcated by the white boxes were amplified in the right panel. tdTomato was primarily detected in the developing renal cortices. Scale bar: 100 μm.

To examine the tissue distribution of Emx1-Cre recombination activity, we crossed Emx1-Cre mice with Ai14 mice (hereafter Emx1-Ai14) (Figure 1A). Ai14 is a Cre reporter tool strain that will express robust tdTomato fluorescence after Cre-mediated recombination (Madisen et al., 2010). As expected, tdTomato fluorescence was detected in the neocortex of E18.5 Emx1-Ai14 embryos (n = 3), whereas little fluorescence was detected in E18.5 Ai14 embryos (n = 3) (Figure 1B). After inspecting the whole E18.5 Emx1-Ai14 embryos, we found that tdTomato fluorescence could be also detected in the kidney region (n = 3). The fluorescence intensity was at a comparable level to that in the neocortex (Figure 1C). To examine tdTomato fluorescence in greater detail, we dissected the kidney from E18.5 Emx1-Ai14 embryos. The fluorescence was primarily localized in the renal cortex but not in the medulla (n = 3) (Figure 1D). This preferential localization in the cortex was more prominent in postnatal day (P) 3 (n = 3) (Figure 1E) as well as P7 kidneys (n = 3) (Figure 1F). We concluded that Emx1-Cre mediates robust recombination in the renal cortex during embryogenesis.

### Emx1-Cre mediates recombination in epithelial cells of proximal and distal convoluted tubules

**FIGURE 2:**
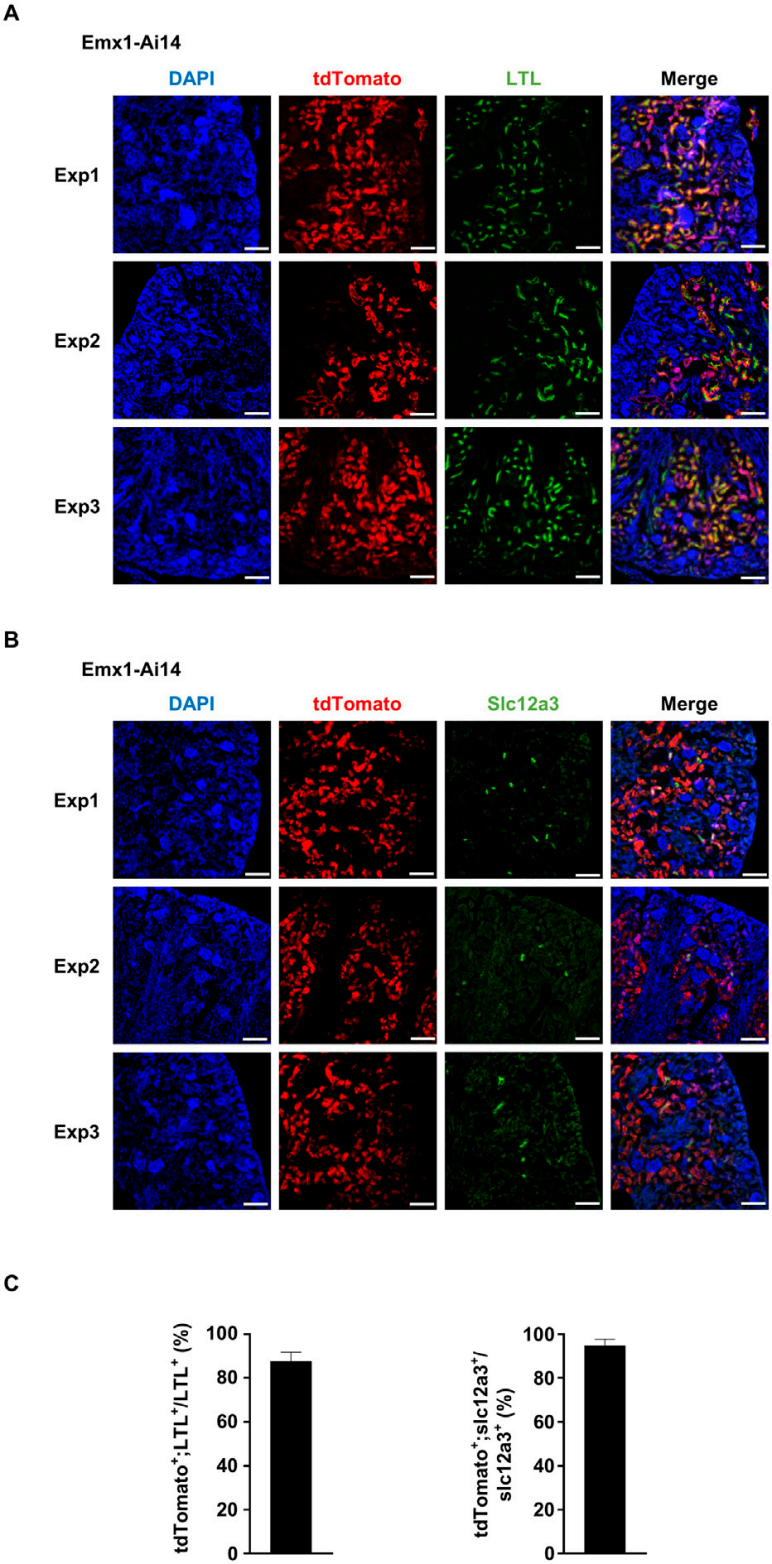
Emx1-Cre mediates recombination in epithelial cells of proximal and distal convoluted tubules **(A)** Immunofluorescence analysis of the colocalization between tdTomato and fluorescein-labeled lotus tetragonolobus lectin (LTL). Representative sagittal sections of P7 Emx1-Ai14 kidney from three independent experiments (Exp1, Exp2 and Exp3) were shown (n = 5). LTL labels epithelial cells in the proximal convoluted tubules. Scale bar: 100 μm. **(B)** Immunofluorescence analysis of the colocalization between tdTomato and Slc12a3. Representative sagittal sections of P7 Emx1-Ai14 kidney from three independent experiments (Exp1, Exp2 and Exp3) were shown (n = 5). Slc12a3 labels epithelial cells in the distal convoluted tubules. Scale bar: 100 μm. **(C)** Quantification of tdTomato^+^;LTL^+^ among LTL^+^ cells, and tdTomato^+^;Slc12a3^+^ among Slc12a3^+^ cells. Three fields from each of Exp1, Exp2 and Exp3 were used for the quantification. The bar plot represents mean ± SD.

By visual inspection, we suspected that tdTomato fluorescence was localized in the proximal and distal tubules. To confirm this observation, five independent experiments were carried out to analyze the colocalization of tdTomato fluorescence with established markers. Kidneys were dissected from P7 Emx1-Ai14 mice. Lotus tetragonolobus lectin (LTL) labels epithelial cells in the proximal convoluted tubules in the renal cortex (Chen et al., 2021). We found that the majority of cells labeled by fluorescein-conjugated LTL also exhibited tdTomato fluorescence (n = 5) (Figures 2A, C). Slc12a3 labels epithelial cells in the distal convoluted tubules (Chen et al., 2021). We found that most Slc12a3-positive cells also exhibited tdTomato fluorescence (n = 5) (Figures 2B, C). These results demonstrated that Emx1-Cre can drive recombination in epithelial cells lining proximal and distal convoluted tubules of the nephron.

### Nestin-Cre mediates recombination in proximal as well as distal convoluted tubules and the glomerulus

**FIGURE 3:**
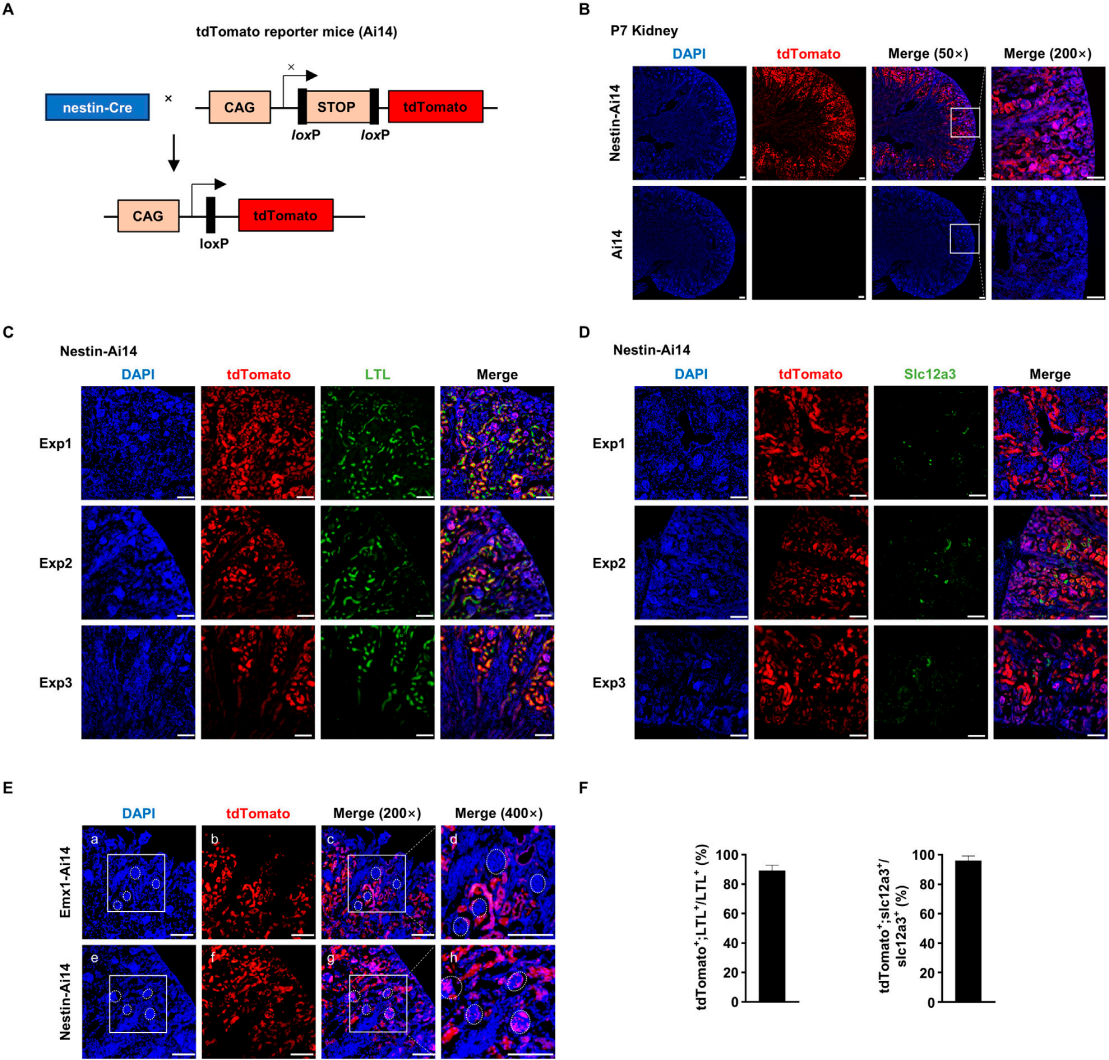
Nestin-Cre mediates recombination in proximal as well as distal convoluted tubules and the glomerulus **(A)** The schematic presentation of using tdTomato reporter mice (Ai14) to detect the tissue-specific expression of nestin-Cre. tdTomato will exhibit red fluorescence in any cells in which nestin-Cre is expressed. CAG, chicken beta-actin promoter. STOP, STOP cassette containing stop codons in all 3 reading frames and a triple poly(A) signal. nestin-Cre mice and Ai14 mice were crossed to generate Nestin-Ai14 mice. **(B)** Sagittal view of dissected postnatal day (P) 7 kidney from Ai14 and Nestin-Ai14 mice. Representative images from three independent experiments were shown (n = 3). Areas demarcated by the white box were amplified in the right panel. tdTomato was primarily detected in the developing renal cortices. Scale bar: 100 μm. **(C)** Immunofluorescence analysis of the colocalization between tdTomato and fluorescein-labeled lotus tetragonolobus lectin (LTL). Representative sagittal sections of P7 Nestin-Ai14 kidney from three independent experiments (Exp1, Exp2 and Exp3) were shown (n = 5). LTL labels epithelial cells in the proximal convoluted tubules. Scale bar: 100 μm. **(D)** Immunofluorescence analysis of the colocalization between tdTomato and Slc12a3. Representative sagittal sections of P7 Nestin-Ai14 kidney from three independent experiments (Exp1, Exp2 and Exp3) were shown (n = 5). Slc12a3 labels epithelial cells in the distal convoluted tubules. Scale bar: 100 μm. **(E)** A side-by-side comparison of Emx1-Cre and nestin-Cre recombination activity in the renal cortex. Four glomeruli, identified by compacted DAPI staining, were circled by white dashed line in P7 Emx1-Ai14 **(a, c, d)** and Nestin-Ai14 kidney **(e, g, h)**, respectively. The area demarcated by the white box in **(a)** and **(e)** is the same as in **(c)** and **(g)**, respectively. Of note, the tdTomato signal is present in glomeruli of the Nestin-Ai14 mice, but absent in Emx1-Ai14 mice. Scale bar: 100 μm. **(F)** Quantification of tdTomato^+^;LTL^+^ among LTL^+^ cells, and tdTomato^+^;Slc12a3^+^ among Slc12a3^+^ cells. Three fields from each of Exp1, Exp2 and Exp3 were used for the quantification. The bar plot represents mean ± SD.

These observations prompted us to ask whether other Cre systems used to study brain development also exhibit recombination activity in the kidney. We crossed nestin-Cre mice with Ai14 mice (hereafter Nestin-Ai14) (Figure 3A). Like in Emx1-Ai14 mice, tdTomato fluorescence was detected primarily in the renal cortex of P7 Nestin-Ai14 kidneys (n = 3) (Figure 3B). In addition, we found that the majority of cells labeled by fluorescein-conjugated LTL also exhibited tdTomato fluorescence (n = 5) (Figures 3C, F), and all Slc12a3-positive cells exhibited tdTomato fluorescence (n = 5) (Figures 3D, F). Finally, we carried out a side-by-side comparison of tdTomato fluorescence in P7 Nestin-Ai14 and Emx1-Ai14 kidneys. We found that tdTomato fluorescence could be detected in the glomerulus of the nephron in P7 Nestin-Ai14, but not in Emx1-Ai14 kidneys (n = 3) (Figure 3E). These results demonstrated that nestin-Cre, like Emx1-Cre, can drive recombination in epithelial cells lining proximal and distal convoluted tubules, but also in the glomerulus of the nephron.

### Knockout of *Larp7* by nestin-Cre, but not Emx1-Cre, results in impaired renal function

**FIGURE 4:**
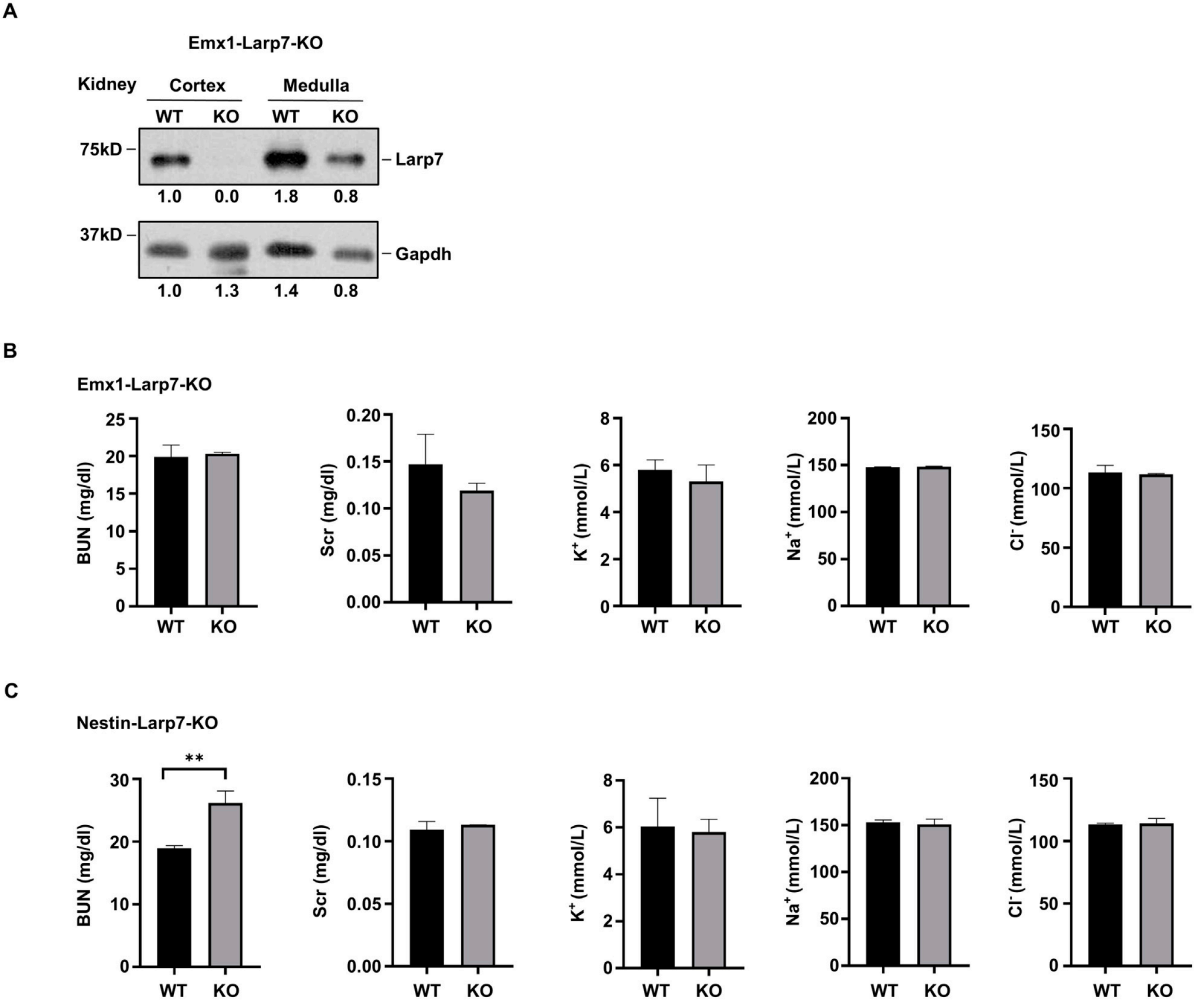
Knockout of *Larp7* by nestin-Cre, but not Emx1-Cre, results in impaired renal function **(A)** Protein blot analysis of indicated proteins in wild-type (WT) and *Larp7* knockout mice. *Larp7* ^*flox/flox*^ mice (WT) were crossed with Emx1-Cre or nestin-Cre to knock out *Larp7*, and homozygous knockout offsprings were denoted as Emx1-Larp7-KO and Nestin-Larp7-KO, respectively. Renal cortex was separated from renal medullar by micro-dissection. Representative blot and the quantification of Larp7 and Gapdh protein levels from three independent experiments was shown. **(B)** Analysis of renal functions in WT and Emx1-Larp7-KO mice. Note that the level of BUN, Scr, K^+^, Na^+^ and Cl^−^ in WT and KO blood were comparable. BUN, blood urea nitrogen. Scr, serum creatinine. The bar plot represents mean ± SD (n = 3). **(C)** Analysis of renal functions in WT and Nestin-Larp7-KO mice. Note that the level of BUN was increased in Nestin-Larp7-KO mice, whereas Scr, K^+^, Na^+^ and Cl^−^ levels were comparable in WT and Nestin-Larp7-KO mice. BUN, blood urea nitrogen. Scr, serum creatinine. The bar plot represents mean ± SD (n = 3). **, *p* < 0.01; unpaired t-test.

In humans, inactivating mutations of *LARP7* have been linked to Alazami syndrome, a human NDD with other comorbidities. We recently generated *Larp7* knockout mice using either Emx1-Cre or nestin-Cre (hereafter Emx1-Larp7-KO and Nestin-Larp7-KO, respectively) to examine the effect of *Larp7* loss on neurodevelopment. In light of findings presented so far, we examined Larp7 protein expression in the kidneys of these mice. The renal cortex and the renal medulla were separately dissected to extract total proteins. In both P90 Emx1-Larp7-KO (n = 3) and Nestin-Larp7-KO kidneys, Larp7 protein levels were greatly reduced in the cortex but not in the medulla (Figure 4A). This result is consistent with the fact that both Emx1-Cre and nestin-Cre can mediate recombination in the renal cortex (Figures 2, 3).

To assess the renal function, blood tests were carried out to measure the levels of blood urea nitrogen (BUN), serum creatinine (Scr), and electrolytes (potassium (K^+^), sodium (Na^+^) and chloride (Cl^−^)). Emx1-Larp7-KO and wild-type mice exhibited comparable levels of these five parameters (Figure 4B). In contrast, Nestin-Larp7-KO mice exhibited increased BUN levels, compared to wild-type littermates. The Scr and electrolyte levels were normal in Nestin-Larp7-KO mice (Figure 4C). Considering that nestin-Cre (but not Emx1-Cre) can mediate recombination in the renal glomeruli (Figure 3D), these observations indicated that under homeostatic conditions, the filtering functions of the nephron may be compromised by *Larp7* genetic ablation.

## Discussion

In this report, we demonstrate that two popular mouse lines, used to study mammalian neurogenesis, can mediate recombination in the renal cortex. Both Emx1-Cre and nestin-Cre exhibit robust activities in epithelial cells lining proximal and distal convoluted tubules of the nephron. The temporal and spatial expression pattern of Emx1-Cre mirrors that of endogenous Exm1 in the brain, because Emx1-Cre was constructed by knocking in an internal ribosome entry site and a Cre recombinase coding sequence to the 3′-untranslated region of Emx1 gene (Gorski et al., 2002). However, little was known about the expression pattern and function of Emx1 gene in the kidney. Recently, Ransick et al. (Ransick et al., 2019) and Chen et al. (Chen et al., 2021) used single-cell sequencing to analyze the transcriptomic profiles of the kidney from 10-week-old mice. Both studies found that Emx1 mRNA is primarily expressed in the distal convoluted tubule region of the nephron in 10-week-old mice. In this study, we found that Emx1-Cre exhibited full activity in both proximal and distal convoluted tubules at E18.5. Taken together, these results indicate that Emx1 is highly expressed in both proximal and distal convoluted tubules during embryogenesis, but its expression becomes gradually restricted to the distal convoluted tubule in the adult. Additionally, nestin-Cre drives recombination in the glomerulus of the nephron. Building upon these findings, we show that *Larp7* may be required for the filtering functions of renal glomeruli. Inactivating mutations of *LARP7* have been linked to Alazami syndrome, a human NDD characterized by severe intellectual disability. Recent studies have also revealed other comorbidities such as kidney abnormalities in Alazami syndrome patients (Fauntleroy-Love et al., 2023). Further studies using Nestin-Larp7-KO mouse model established in this study may provide mechanistic insight on etiologies of Alazami syndrome patients.

One intriguing implication of our study is that some NDDs may have a renal connection. This assumption is supported by the fact that in human patients with chronic kidney disease, many exhibit cognitive impairment and deficits in learning, memory, and sensory processing (Zoccali et al., 2017). Dozens of genes have been knocked out using Emx1-Cre and nestin-Cre to study their contributions in neurodevelopment. It will be of great interest to reinvestigate these mouse models whether the renal function is impaired. And if so, how compromised renal function impairs neurogenesis.

## Materials and methods

### Mice

Emx1-Cre (B6.129S2-Emx1^tm1(cre)Krj^/J; Jackson Lab, #005628) and nestin-Cre (B6. Cg-Tg (Nes-cre) 1Kln/J; Jackson Lab, #003771) mice were previously described, and obtained from Cyagen Biosciences (Suzhou, China). To identify tissue expression patterns of Emx1-Cre and nestin-Cre, they were crossed with Ai14(B6.Cg-Gt(ROSA)26Sor^tm14(CAG-tdTomato)Hze^/J; Jackson Lab, #007914) reporter mice. In Ai14 reporter mice, a 3X STOP codons flanked by two *lox*P sites are localized upstream of the tdTomato coding sequence, thus preventing tdTomato expression without Cre recombinase activity. To generate conditional knockout of *Larp7*, *Larp7* ^*flox/flox*^ mice were crossed with Emx1-Cre or nestin-Cre mice, respectively. Noon on the day of the vaginal plug was defined as E0.5. Embryos and offsprings were genotyped by PCR of genomic DNA. The following primers are used: Genome-Ai14-F: GGC​ATT​AAA​GCG​CTA​TCC; Genome-Ai14-R: CTG​TTC​CTG​TAC​GGC​ATG​G; Genome-Cre-F: ACC​CTG​TTA​CGT​ATA​GCC​GA; Genome-Cre-R: CTC​CGG​TAT​TGA​AAC​TCC​AG; Genome-Larp7-floxed-F: ATG​TAT​CAG​CTC​TGG​GGA​AAG​TAG; Genome-Larp7-floxed-R: GTA​TCT​TCA​AAC​AGC​TAG​GGC​TCC.

### Immunofluorescence staining, image analysis and protein analysis

Samples were fixed with 4% paraformaldehyde overnight at 4°C, and cryopreserved in 25% sucrose for 1 day before embedding in Tissue-Tek O.C.T. Compound (Sakura Finetek, USA). Samples were sectioned on a cryostat at 8 μm. The glass slides were washed three times (5 min each time) with phosphate buffer saline (PBS), and incubated with blocking solution containing 0.1% Triton X-100% and 10% fetal bovine serum (FBS) for 1 h at room temperature. Then, slides were incubated at 4°C overnight with fluorescein-labeled lotus tetragonolobus lectin (LTL) (Vector Laboratory, FL-1321) to detect proximal convoluted tubules, and with anti-Slc12a3 (Abcam, AB95302) to detect distal convoluted tubules. The next day, slides were washed in PBS for three times (5 min each time), incubated with donkey-anti-rabbit secondary antibodies (Abcam, ab150073) at room temperature for 2 h, and then counterstained by 4′,6-diamidino-2-phenylindole (DAPI) for 10 min. Finally, slides were washed three times with PBS before mounting with Fluoromount (Southern Biotech, 0100-01). Images were documented by inverted fluorescence microscopy (DMi8, Leica, Germany), and analyzed by Leica Application Suite X. The percentage of tdTomato^+^;LTL^+^ among LTL^+^ cells, and tdTomato^+^;Slc12a3^+^ among Slc12a3^+^ cells were quantified by ImageJ. For protein blotting, P90 WT and Emx1-Larp7 KO mice were used. The renal cortex was separated from medulla by micro-dissection, grinded by cryogenic grinder (Ningbo Xinyi ultrasonic equipment Co., Ltd, Xlnyl-24N) in RIPA buffer (Sigma, R0278), supplemented with 0.5 mM DTT, 0.1% PMSF, 5 mM NaF, 2 mM Na_3_VO_4_, cleared by centrifugation at 4°C (13,000 *g*, 10 min), and the supernatant was removed for analysis. The specificity of anti-Larp7 polyclonal antibodies were previously verified (Dai et al., 2014).

## Analysis of renal functions

Blood was obtained from the retro-orbital plexus of mice in deep anesthesia, with the use of micro-hematocrit tubes (Hirschmann, 901618). Blood urea nitrogen (BUN) levels were measured in individual samples using the urea nitrogen reagents (Siemens, 03040257). Serum creatinine (Scr) levels were measured using the Enzymatic Creatinine_2 Reagents (Siemens, 04992596). The levels of Na^+^, K^+^, Cl^−^ were tested using ion selective electrode method. Data was automatically collected by ADVIA chemistry XPT.

### Statistical analyses

The values of each parameter within a group were expressed as the mean ± SD. For comparisons between groups with normally distributed data, statistical significance was assessed using the two-tailed, unpaired t-test. *p* < 0.05 were regarded as statistically significant.

## Data Availability

All data needed to evaluate the conclusions in the paper are present in the paper. Additional data related to this paper may be requested from the lead corresponding author.
